# Effects of Remimazolam Anesthesia in Patients Undergoing Deep Inferior Epigastric Perforator Flap Reconstruction for Breast Cancer: A Retrospective Cohort Study

**DOI:** 10.7150/ijms.125732

**Published:** 2026-04-08

**Authors:** Myoung Hwa Kim, Byongnam Jun, So Yeon Kim, Lin Lin, Hye Jung Shin, Dong Won Lee, Na Young Kim

**Affiliations:** 1 Department of Anesthesiology and Pain Medicine, Anesthesia and Pain Research Institute, Yonsei University College of Medicine, Gangnam Severance Hospital, 211 Eonju-ro, Gangnam-gu, Seoul 06273, Republic of Korea.; 2 Department of Anesthesiology and Pain Medicine, Anesthesia and Pain Research Institute, Yonsei University College of Medicine, 50-1 Yonsei-ro, Seodaemun-gu, Seoul 03722, Republic of Korea.; 3 Department of Anesthesiology and Pain Medicine, Yonsei University College of Medicine, Seoul 03722, Republic of Korea.; 4 Biostatistics Collaboration Unit, Yonsei University College of Medicine, 50-1 Yonsei-ro, Seodaemun-gu, Seoul 03722, Republic of Korea.; 5 Department of Plastic and Reconstructive Surgery, Institute for Human Tissue Restoration, Yonsei University College of Medicine, 50-1 Yonsei-ro, Seodaemun-gu, Seoul 03722, Republic of Korea.

**Keywords:** breast reconstruction, hemodynamic stability, propofol, remimazolam, sevoflurane

## Abstract

In deep inferior epigastric perforator (DIEP) free flap surgery for breast reconstruction, vasopressors may cause vasoconstriction and impair flap perfusion, potentially increasing the risk of complications. In this study, we aimed to determine the effects of remimazolam anesthesia on intraoperative vasopressor requirements and postoperative outcomes in patients undergoing DIEP flap reconstruction compared with conventional anesthesia. We conducted a retrospective cohort study of DIEP flap procedures performed between March 2021 and March 2022 at a single institution. Patients were stratified according to the anesthetic regimen (sevoflurane, propofol, and remimazolam). The primary outcome was the total intraoperative norepinephrine requirement. Among 102 initially identified patients, 75 met the inclusion criteria. The remimazolam group required significantly less norepinephrine than the sevoflurane and propofol groups (202.1 µg vs. 1558.6 µg and 926.6 µg, respectively; *P <* 0.001), with comparable reductions in ephedrine use. Thus, remimazolam may serve as an effective alternative to sevoflurane and propofol anesthesia in terms of reducing intraoperative vasopressor requirements and is potentially better in maintaining hemodynamic stability during DIEP free flap reconstruction.

## 1. Introduction

The transverse rectus abdominis musculocutaneous and deep inferior epigastric perforator (DIEP) flaps are the two most common free flap techniques used for breast reconstruction 1. Unlike the transverse rectus abdominis musculocutaneous flap, the DIEP flap preserves abdominal wall integrity by sparing the rectus abdominis muscle, reducing hernia risk by 50% and shortening the recovery time 2. Therefore, the DIEP flap is considered the gold standard for autologous breast reconstruction 2-4.

Despite its advantages, such as low complication rates and natural aesthetic outcomes, DIEP flap surgery remains technically complex and time-intensive, presenting unique anesthetic challenges 5. Maintaining optimal flap perfusion is paramount, as inadequate blood supply can lead to complications such as flap loss or fat necrosis 6. Intraoperative management combines vasopressor and fluid administration to maintain optimal blood pressure and blood flow, thereby preserving flap viability during flap grafting surgery 7. However, vasopressor use may induce vasoconstriction and compromise flap perfusion 6. Therefore, the ideal anesthetic regimen for DIEP flap procedures requires a balance between hemodynamic stability and minimal vasopressor dependence.

Recently, remimazolam, an ultra-short-acting benzodiazepine, has been introduced for both induction and maintenance of general anesthesia 8-10. It offers several advantages over conventional benzodiazepines, including rapid onset, short context-sensitive half-life, metabolism via tissue esterases (primarily liver carboxylesterase), inactive metabolites, availability of an antidote (flumazenil), and excellent hemodynamic stability 11, 12. These properties make remimazolam suitable for continuous intravenous infusion, and it is increasingly used for both total intravenous anesthesia and procedural sedation.

We hypothesized that remimazolam-remifentanil anesthesia would provide better hemodynamic stability than conventional anesthetic methods (sevoflurane or propofol) in patients undergoing DIEP flap reconstruction. In this study, we aimed to compare the intraoperative vasopressor requirements and postoperative recovery outcomes among three anesthetic regimens: (1) sevoflurane-remifentanil, (2) propofol-remifentanil, and (3) remimazolam-remifentanil.

## 2. Materials and Methods

### Patients

This retrospective cohort study was approved by the Institutional Review Board of the Yonsei University Health System, Seoul, Republic of Korea (IRB protocol No. 4-2022-1159). The requirement for informed consent was waived because of the retrospective study design. This study was conducted in accordance with the Declaration of Helsinki. We reviewed the electronic medical records of 102 consecutive patients who underwent unilateral mastectomy with immediate DIEP free flap reconstruction for breast cancer at the Yonsei Cancer Center between March 2021 and March 2022. The exclusion criteria were as follows: (1) age > 70 years, to minimize potential confounding from age-associated comorbidities; (2) use of desflurane or combined anesthetic regimens; (3) concurrent surgical procedures; and (4) incomplete medical records.

### Anesthesia and Surgical Procedures

Upon arrival in the operating room, the patients were monitored using electrocardiography, pulse oximetry, and noninvasive blood pressure measurement. Additional monitoring included SedLine^®^ electroencephalography (Masimo Corp., Irvine, CA, USA) for Patient State Index measurement and a peripheral nerve stimulator for neuromuscular blockade assessment. Premedication with 0.1 mg glycopyrrolate was administered prior to anesthesia induction. After loss of consciousness, 0.6-1.0 mg/kg of rocuronium was administered to facilitate endotracheal intubation, followed by continuous infusion to maintain a train-of-four count of 0-2. The mechanical ventilation parameters included a tidal volume of 7-8 mL/kg, 50% fraction of inspired oxygen, positive end-expiratory pressure of 5 cmH_2_O, and target end-tidal carbon dioxide of 35-40 mmHg. All patients underwent radial artery cannulation for continuous arterial pressure monitoring and internal jugular vein central venous catheterization, not only for central venous pressure monitoring but also to ensure a reliable route for the continuous infusion of potent vasopressors such as norepinephrine during the prolonged surgical duration. Hypotension was defined as a mean arterial pressure (MAP) < 60 mmHg and was managed with 4-mg boluses of ephedrine or a continuous norepinephrine infusion. Body temperature was maintained at 36-37 °C using a forced-air warming system.

The attending anesthesiologist titrated the anesthetic drugs to maintain a Patient State Index between 25 and 50. All anesthetic agents for target-controlled infusion (TCI) were administered using a commercial infusion pump (Orchestra® Base Primea, Fresenius-Kabi, Sèvres, France). The attending anesthesiologist selected the anesthetic regimen based on clinical preference and not according to a predefined study protocol or specific patient characteristics. In the sevoflurane group, induction was achieved with a bolus dose of 1.0-1.5 mg/kg of propofol combined with a TCI of remifentanil at an effect site concentration (Ce) of 4.0 ng/mL. Anesthesia was maintained using age-adjusted end-tidal sevoflurane (0.8-1.0 minimal alveolar concentration) with continuous remifentanil TCI. In the propofol group, both induction and maintenance were accomplished using TCI of propofol (Ce 4.0-4.5 μg/mL) and remifentanil (Ce 4.0 ng/mL). In the remimazolam group, induction was performed with remimazolam (Byfavo^®^; Hana Pharmaceutical Co., Ltd., Seoul, Korea) infused at 6 mg/kg/h with remifentanil TCI (Ce 4.0 ng/mL), followed by maintenance with remimazolam at 1-2 mg/kg/h and continued remifentanil TCI.

Following induction, patients were positioned slightly upright before being returned to the supine position for mastectomy completion. The supine position was maintained throughout flap harvesting, detachment, and microsurgical revascularization. After vessel anastomosis and flap reperfusion, the patients were carefully repositioned in a sitting (beach chair) position to evaluate breast aesthetics and symmetry while reducing tension on the abdominal donor site. This position was maintained during anesthetic emergence and throughout the postoperative recovery period. Prior to surgery completion, all patients received intravenous oxycodone (0.08 mg/kg total dose) for analgesia and ramosetron (0.3 mg, IV) for postoperative nausea and vomiting prophylaxis. Regional anesthesia techniques such as pectoral nerve blocks or transversus abdominis plane blocks were not performed. This protocol was adopted to prevent potential tissue edema or hematoma at the abdominal donor site that could interfere with perforator dissection and to strictly isolate the hemodynamic effects of systemic anesthetic agents.

Following surgery completion, neuromuscular blockade was reversed using sugammadex (Bridion®, MSD, Seoul, Korea). All anesthetic agents were discontinued, and the patients in the remimazolam group received 0.2-0.5 mg flumazenil (Flunil^®^; Bukwang Pharmaceutical Co., Ltd., Seoul, Korea) for benzodiazepine reversal. Upon confirmation of spontaneous eye opening and an appropriate response to verbal commands, the endotracheal tube was extubated. The patients were then transferred to the post-anesthesia care unit (PACU), where recovery management was performed under physician supervision. After a mandatory 30-min observation period and achievement of an Aldrete score ≥ 9, patients were discharged from the PACU.

### Outcomes Assessment and Data Collection

The primary outcome measure was the total intraoperative norepinephrine requirement in the study groups. Comprehensive baseline patient characteristics were collected through medical record review, including demographic data (age, sex, and body mass index), American Society of Anesthesiologists physical status, comorbidities, smoking history, menopausal status, neoadjuvant chemotherapy exposure, and preoperative tumor markers (carcinoembryonic antigen and cancer antigen). Tumor characteristics included hormone receptor status, human epidermal growth factor receptor status, histopathological type and grade, and clinical staging.

Detailed intraoperative records included temporal parameters (anesthesia duration, reconstruction time, and total operative time), anesthetic consumption (total remifentanil, propofol, and remimazolam administered), vasopressor requirements (ephedrine and norepinephrine doses), and fluid balance data (total crystalloid/colloid administration, estimated blood loss, and urine output). Transfusion events during the surgery were also recorded. Surgical parameters included mastectomy type, lymph node management approach, tumor dimensions, and specimen weights (both breasts and harvested flap).

Perioperative hemodynamic variables, including MAP and heart rate (HR), were recorded at eight defined time points: pre-induction (baseline), 10 min post-intubation, mastectomy completion, initiation of microscopic anastomosis/ revascularization, 30 min into microscopic anastomosis/revascularization, completion of microscopic anastomosis/revascularization, 10 min after beach chair positioning, and procedure completion. Pulse pressure variation (PPV) was measured at all time points, except for the baseline.

PACU data included the duration of PACU stay, numeric rating scale pain scores, rescue fentanyl requirements, and metoclopramide administration for postoperative nausea/vomiting. The postoperative hospital length of stay and adjuvant treatment initiation were recorded. Surgical complications within 48 h were recorded, including transfusion requirements, reoperation, flap necrosis/detachment, hematoma formation, wound dehiscence, and venous congestion. Surgical reoperation was indicated by specific clinical signs of compromised perfusion, including sudden tense swelling of the flap, venous congestion, or deterioration of Doppler signals, rather than mild swelling alone.

### Statistical Analysis

Continuous variables are presented as means ± standard deviations or median (interquartile range), whereas categorical variables are expressed as frequency (percentage). We assessed data normality using the Shapiro-Wilk test. Normally distributed continuous variables were compared among groups using one-way analysis of variance with post-hoc *t*-tests, whereas non-normally distributed variables were analyzed using the Kruskal-Wallis test with post-hoc Mann-Whitney U tests. Categorical variables were compared using chi-square tests, with Fisher's exact test applied when > 20% of cells had expected frequencies < 5. For repeated measures (MAP, HR, and PPV), a linear mixed model with a compound symmetry covariance structure was employed to account for within-subject effects. This model estimated the least square means and standard errors for each group at all time points. Primary comparisons evaluated significant differences between (1) sevoflurane vs. remimazolam and (2) propofol vs. remimazolam groups at each time point, with Bonferroni correction for multiple comparisons.

All analyses were performed using SAS 9.4 (SAS Institute, Cary, NC, USA). Statistical significance was set at two-tailed *P <* 0.05, with adjusted thresholds of *P* < 0.017 (0.05/3) for three comparisons and *P* < 0.025 (0.05/2) for two comparisons, after Bonferroni correction.

## 3. Results

**Figure 1:**
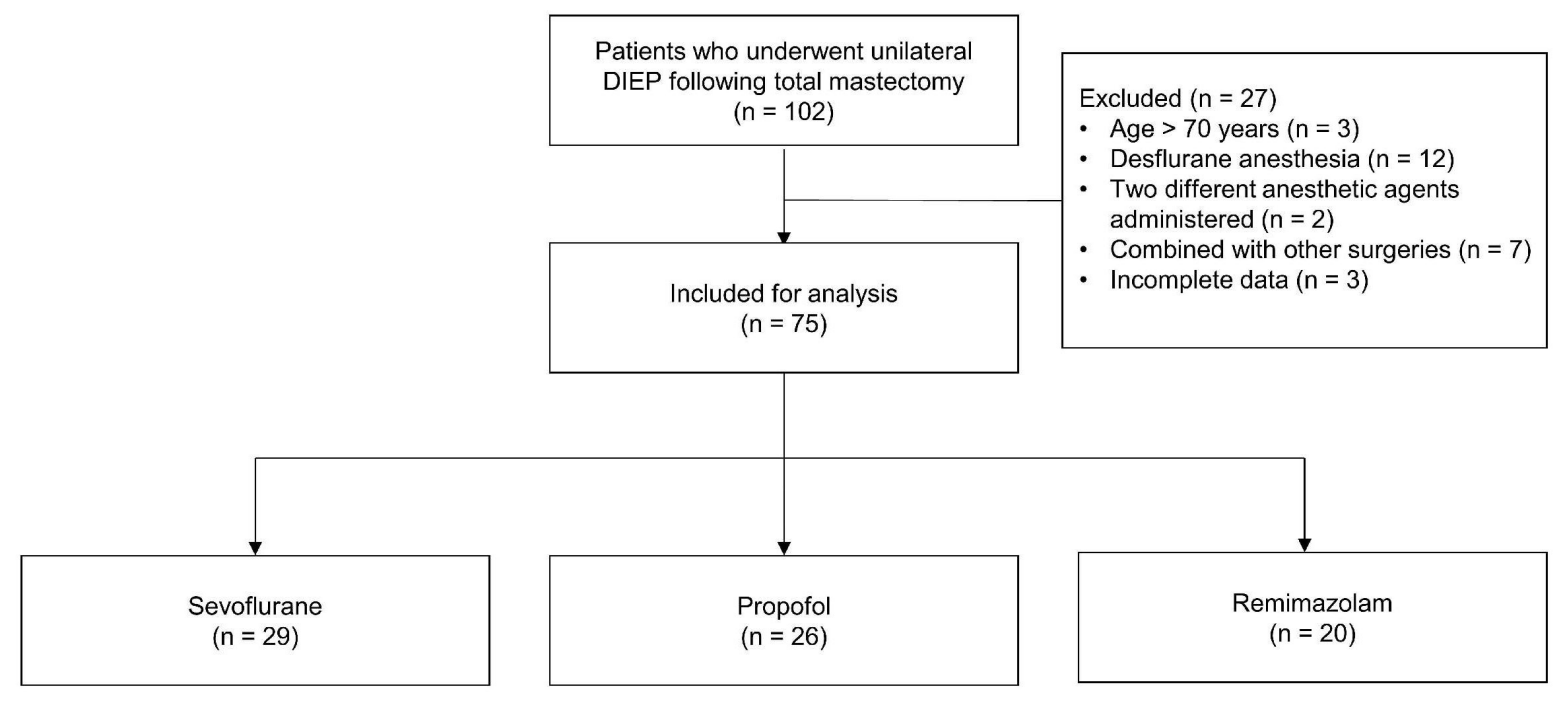
Flow diagram. **Abbreviations:** DIEP: deep inferior epigastric perforator.

The study flow diagram is presented in Figure 1. Of the initial 102 patients, 27 were excluded based on the following criteria: age > 70 years (n = 3), desflurane use (n = 12), combined anesthetic regimens (n = 2), concurrent surgical procedures (n = 7), and incomplete medical records (n = 3). The final analysis included 75 patients distributed across three groups: sevoflurane (n = 29), propofol (n = 26), and remimazolam (n = 20).

**Figure 2:**
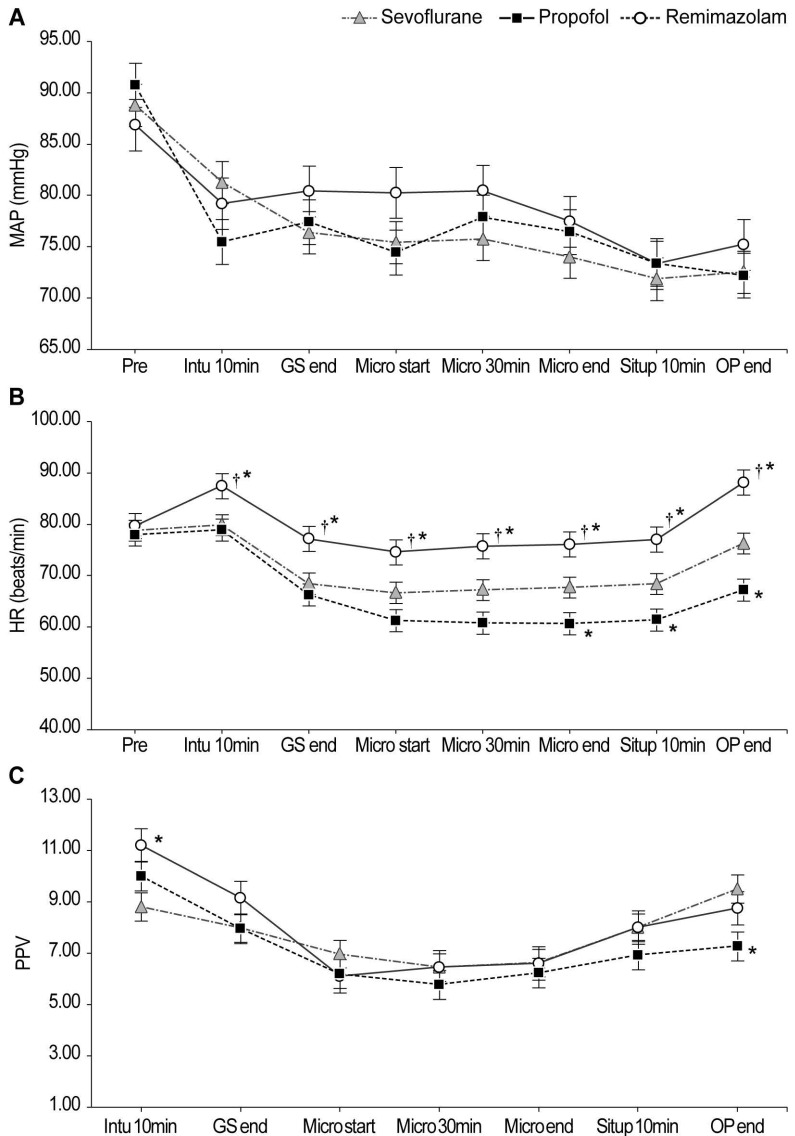
Intraoperative hemodynamic changes. The linear mixed-model analysis showed significant group-by-time interaction effects in HR (*P <* 0.001) and PPV (*P =* 0.014). ^*^Bonferroni-corrected *P <* 0.05/2 compared with the sevoflurane group. ^†^Bonferroni-corrected *P <* 0.05/2 compared to the propofol group. **Abbreviations:** MAP: mean arterial pressure; HR: heart rate; PPV: pulse pressure variation; Pre: pre-induction; Intu 10min: 10 min after intubation; GS end: at the end of mastectomy; Micro start: at the start of microscopic re-anastomosis and revascularization; Micro 30min: 30 min into microscopic re-anastomosis and revascularization; Micro end: at the end of microscopic re-anastomosis and revascularization; Situp 10min: 10 min after steep sit-up positioning; OP end: at the end of the operation.

**Figure 3:**
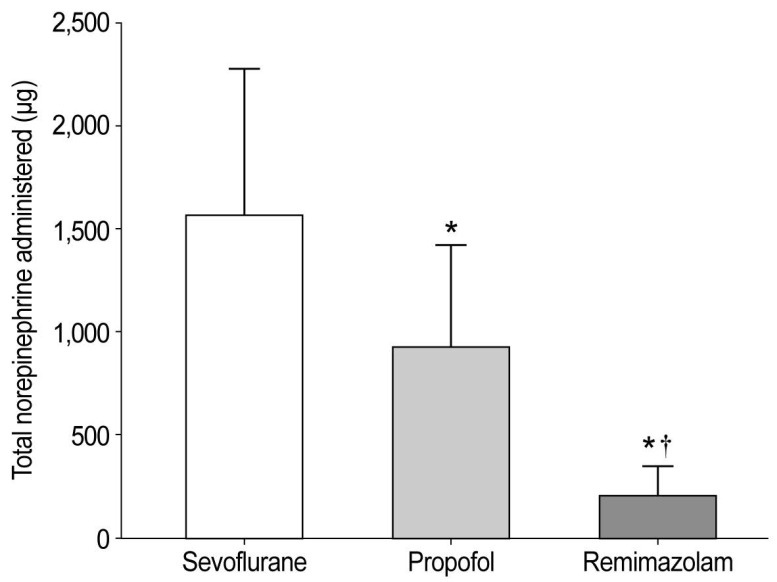
Total intraoperative norepinephrine consumption. The total amount of norepinephrine administered was 1558.6 ± 718.2 µg in the sevoflurane group, 926.6 ± 494.5 µg in the propofol group, and 202.1 ± 144.4 µg in the remimazolam group (*P <* 0.001). ^*^Bonferroni-corrected *P <* 0.05/2 compared with the sevoflurane group. ^†^Bonferroni-corrected *P <* 0.05/2 compared to the propofol group.

Baseline demographic characteristics showed no significant intergroup differences (Table 1). The intraoperative parameters are summarized in Table 2. Intraoperative hemodynamic changes are shown in Figure 2. Significant interaction effects were observed in HR (*P <* 0.001) and PPV (*P =* 0.014) but not in MAP. HR remained significantly higher in the remimazolam group than in the other groups throughout anesthesia. Notably, total norepinephrine consumption was lowest in the remimazolam group (202.1 µg), significantly lower than that in the propofol (926.6 µg) and sevoflurane (1,558.6 µg) groups (Figure 3).

The postoperative recovery profiles are summarized in Table 3. In terms of raw incidence, adverse events such as reoperation, flap detachment, hematoma, and venous congestion were most frequent in the sevoflurane group, whereas the remimazolam group exhibited the lowest rate. However, no statistically significant differences were observed in individual complications among the three groups.

## 4. Discussion

In this study, we compared the intraoperative hemodynamic changes and vasopressor requirements in patients undergoing DIEP flap reconstruction for breast cancer using sevoflurane, propofol, or remimazolam. The use of vasopressors, including ephedrine and norepinephrine, was significantly lower in the remimazolam group. To our knowledge, this is the first study to evaluate the perioperative environment in breast cancer free-flap surgery by comparing remimazolam with conventional anesthetic agents.

Understanding factors associated with flap failure or postoperative complications is essential for risk mitigation 13. Among these, intraoperative catecholamine administration remains controversial 7, 14. While catecholamines are often necessary to counteract anesthetic-induced vasodilation, they may impair tissue perfusion through vasoconstriction 7. Thus, many surgeons aim to minimize vasopressor use during free-flap procedures. However, the hemodynamic effects of different anesthetic agents and their impact on vasopressor requirements in microvascular breast reconstruction remain inadequately studied.

During breast reconstruction, patients are frequently repositioned in a sitting position to assess breast symmetry 5. This maneuver can cause hemodynamic instability due to venous pooling, requiring additional vasopressors and fluid boluses 5. Furthermore, abdominal free-flap reconstruction is more complex and time-consuming than implant-based reconstruction, increasing intraoperative fluid and vasopressor requirements 1. Our findings suggest that the choice of anesthetic agent may reduce these requirements.

Total remifentanil consumption was significantly higher in the remimazolam group. This reflects the characteristic of remimazolam-based anesthesia, which requires higher opioid support than volatile agents to maintain adequate anesthetic depth. From a pharmacological perspective, high-dose remifentanil typically induces dose-dependent hypotension and bradycardia 15. Paradoxically, despite the higher opioid-induced hypotensive effects, the remimazolam group required the least vasopressor support. This finding strongly reinforces the superior hemodynamic stability of remimazolam, suggesting its ability to preserve vascular tone even under high-opioid conditions.

There is no clear consensus on whether inhalational or intravenous anesthesia is superior for flap-based breast reconstruction 16. Conventional agents, such as sevoflurane and propofol, cause vasodilation and hypotension, particularly in high-risk patients 16. Conversely, remimazolam, an ultra-short-acting benzodiazepine, has expanded the options for total intravenous anesthesia 17, 18. At standard doses, remimazolam causes minimal cardiovascular and respiratory depression and less hypotension than propofol 19-21. Its hemodynamic stability may result from dose-dependent increases in intracellular calcium via the G protein-coupled receptor-inositol 1,4,5-triphosphate pathway 22.

Regarding the heart rate profile, the remimazolam group exhibited consistently higher heart rate values compared to the propofol and sevoflurane groups. This finding likely reflects a preserved baroreceptor reflex rather than insufficient anesthetic depth, as the Patient State Index was maintained between 25 and 50 in all patients. Unlike propofol, which blunts autonomic reflexes and induces bradycardia, remimazolam preserves baroreflex sensitivity, allowing for physiological heart rate compensation to maintain cardiac output 23.

The remimazolam group required significantly less vasopressor and fluid support while maintaining the most stable heart rate. Although postoperative complication rates did not differ significantly among the groups, minimizing vasopressor requirements may still be clinically important in free flap surgery. While the relationship between vasopressor uses and flap outcomes remains controversial 7, 14, 24, maintaining hemodynamic stability without excessive vasopressor support represents a theoretical advantage by potentially preserving microvascular perfusion. The substantial reduction in vasopressor requirements observed with remimazolam while maintaining hemodynamic stability may be particularly valuable in prolonged microsurgical procedures, where optimal tissue perfusion is critical.

The present study has some limitations. First, owing to the single-center, retrospective, observational design, the study is susceptible to bias and other confounding factors. Second, the choice of anesthetic regimen was not randomized but was determined by the attending anesthesiologist's preference. This lack of standardization introduces the potential for selection bias, as unrecorded patient characteristics or anticipated surgical difficulties may have influenced anesthetic selection. Third, despite no statistical differences, several operative variables showed trends to reflect differences in surgical complexity, which may have influenced fluid requirements, blood loss, and hemodynamic stability. Further research is needed to completely rule out the possibility that this biased the results in favor of remimazolam. Fourth, as noted in the results, the varying dosage of remifentanil across groups (significantly higher in the remimazolam group) acted as a confounding factor that complicated the isolation of the pure effects of the hypnotic agents. Fifth, we relied on surrogate markers such as vasopressor requirements and clinical outcomes to assess flap perfusion, as direct microcirculatory monitoring (e.g., laser Doppler flowmetry or indocyanine green angiography) was not routinely performed. Finally, the relatively small sample size limited the statistical power to detect significant differences in rare postoperative complications, increasing the risk of type II errors. Therefore, our findings regarding the complication rates should be interpreted as preliminary clinical trends rather than confirmatory evidence. Future large-scale randomized controlled trials are required to validate these results.

## 5. Conclusions

Our findings suggest that remimazolam reduces intraoperative vasopressor requirements compared with sevoflurane and propofol when used for DIEP free flap reconstruction. Thus, remimazolam may be beneficial for patients undergoing prolonged microsurgical procedures, where maintaining hemodynamic stability is critical for optimal tissue perfusion.

## Ethics Approval

The study was conducted in accordance with the Declaration of Helsinki and approved by the Institutional Review Board of the Yonsei University Health System, Seoul, Republic of Korea (IRB protocol No. 4-2022-1159). The requirement for patient consent was waived owing to the retrospective study design.

## Figures and Tables

**Table 1:** Patient characteristics

Variables	Sevoflurane (n = 29)	Propofol (n = 26)	Remimazolam (n = 20)	*P* value
Age, year	49 ± 7	48 ± 8	50 ± 6	0.617
Body mass index (kg/m^2^)	24.4 ± 3.8	23.9 ± 3.6	23.9 ± 2.8	0.848
ASA physical status				0.481
I-II	27 (93%)	26 (100%)	19 (95%)	
III	2 (7%)	0 (0%)	1 (5%)	
Comorbidities				
Hypertension	3 (10%)	3 (12%)	2 (10%)	> 0.999
Diabetes	1 (3%)	0 (0%)	1 (5%)	0.728
Hepatitis	0 (0%)	1 (4%)	0 (0%)	0.613
Smoking history				0.174
None	29 (100%)	23 (88%)	18 (90%)	
Previous	0 (0%)	3 (12%)	2 (10%)	
Postmenopausal status	11 (38%)	7 (27%)	7 (35%)	0.677
Preoperative chemotherapy	9 (31%)	7 (27%)	6 (30%)	0.943
Tumor pathology				0.105
Ductal carcinoma in situ	6 (21%)	8 (31%)	9 (45%)	
Invasive ductal carcinoma	17 (58%)	8 (31%)	8 (40%)	
Infiltrating other	6 (21%)	10 (38%)	3 (15%)	
Histopathologic grade				0.384
Low	12 (41%)	11 (42%)	7 (35%)	
Intermediate	12 (41%)	9 (35%)	12 (60%)	
High	5 (18%)	6 (23%)	1 (5%)	
Stage				0.242
0	6 (21%)	9 (35%)	2 (10%)	
1	9 (31%)	4 (15%)	4 (20%)	
2	13 (45%)	13 (50%)	14 (70%)	
3	1 (3%)	0 (0%)	0 (0%)	

Values are presented as mean ± standard deviation or number of patients (percentage). **Abbreviations:** ASA: American Society of Anesthesiologists

**Table 2:** Intraoperative parameters

Variables	Sevoflurane (n = 29)	Propofol (n = 26)	Remimazolam (n = 20)	*P* value
Intraoperative variables				
Duration of anesthesia (min)	584 ± 70	556 ± 49	583 ± 51	0.112
Duration of reconstruction (min)	399 ± 59	366 ± 48	396 ± 51	0.052
Duration of total operation (min)	538 ± 70	507 ± 47	532 ± 53	0.099
Administered dose of remifentanil (mg)	1.6 ± 0.3	3.0 ± 0.6^*^	3.8 ± 1.3^*^	< 0.001
Administered dose of ephedrine (mg)	6 (0, 12)	8 (0, 16)	0 (0, 2)^*,†^	0.002
Fluid intake (mL)	3,812 ± 725	3,627 ± 684	3,187 ± 819^*^	0.016
Urine output (mL)	1,274 ± 487	1,418 ± 451	816 ± 419^*,†^	< 0.001
Estimated blood loss (mL)	175 ± 93	111 ± 57^*^	108 ± 61^*^	0.006
Intake-output difference (mL)	2,405 ± 698	2,098 ± 672	2,263 ± 715	0.267
Patients transfused during surgery	1 (3%)	1 (4%)	0 (0%)	> 0.999
Surgical information				
Type of mastectomy				0.853
Nipple sparing mastectomy	15 (52%)	15 (58%)	10 (50%)	
Skin sparing mastectomy	14 (48%)	11 (42%)	10 (50%)	
Lymph node procedure				0.670
SLNB only	21 (72%)	20 (77%)	13 (65%)	
SLNB then ALND	8 (28%)	6 (23%)	7 (35%)	
Specimen weight (g)	515 ± 199	501 ± 217	456 ± 210	0.612
Flap weight (g)	507 ± 177	471 ± 173	392 ± 147	0.069
Tumor size (cm)	2.3 ± 1.6	2.7 ± 2.3	3.3 ± 2.1	0.298

Values are shown as mean ± standard deviation or number of patients (percentage), except for the administered dose of ephedrine (median [interquartile range]). ^*^Bonferroni-corrected *P <* 0.05/2 compared to the sevoflurane group. ^†^Bonferroni-corrected *P <* 0.05/2 compared with the propofol group. **Abbreviations:** SLNB: sentinel lymph node biopsy; ALND: axillary lymph node dissection

**Table 3:** Postoperative recovery profiles

Variables	Sevoflurane (n = 29)	Propofol (n = 26)	Remimazolam (n = 20)	*P* value
PACU profile				
Duration of PACU stay (min)	49 ± 23	52 ± 17	48 ± 21	0.812
Numeric rating scale (pain)	3.1 ± 0.4	3.2 ± 0.4	3.0 ± 0.7	0.581
Patients who received fentanyl	3 (10%)	1 (4%)	4 (20%)	0.108
Patients who received metoclopramide	8 (28%)	1 (4%)	2 (10%)	0.035
Ward profile				
Postoperative hospital stays (days)	7.8 ± 1.3	7.4 ± 1.0	7.3 ± 1.4	0.339
Postoperative adjuvant treatment				
Chemotherapy	5 (17%)	3 (12%)	7 (35%)	0.128
Radiotherapy	11 (38%)	7 (27%)	7 (35%)	0.677
Immunotherapy	6 (21%)	3 (12%)	1 (5%)	0.291
Hormone therapy	15 (52%)	19 (73%)	16 (80%)	0.082
Postoperative complications				
Reoperation	4 (14%)	1 (4%)	1 (5%)	0.492
Flap detachment	2 (7%)	0 (0%)	0 (0%)	0.332
Hematoma	3 (10%)	1 (4%)	1 (5%)	0.731
Wound dehiscence	1 (3%)	1 (4%)	0 (0%)	> 0.999
Venous congestion	4 (14%)	1 (4%)	0 (0%)	0.210

Values are presented as the mean ± standard deviation or number of patients (percentage). **Abbreviations:** PACU: postanesthetic care unit

## Data Availability

The research data are available from the corresponding author upon reasonable request. The data are not publicly available due to patient privacy and ethical restrictions.

## Abbreviations

DIEP: deep inferior epigastric perforator
MAP: mean arterial pressure
TCI: target-controlled infusion
PACU: post-anesthesia care unit
HR: heart rate
PPV: pulse pressure variation**.**
